# Round the Hospitals

**Published:** 1919-06-21

**Authors:** 


### ROUND THE HOSPITALS.

A NOTABLE Investiture, at which Admiral of the Fleet Sir David Beatty and Field-Marshal Sir Douglas Haig were invested by the King with the Insignia of the Order of Merit, took place on June 12 in the Quadrangle of Buckingham Palace. His Majesty conferred decorations on members of the Nursing Services as follows: Bar to the Royal Red Cross: Matron Alice Caslim, Q.A.I.M.N.S.R. Royal Red Cross, First Class: Matron Susan Wooler, Q.A.I.M.N.S.; Sister Helen Cox, T.F.N.S.; Sister Maud Francis and Sister Stella Jenkins, Canadian A.N.S. Royal Red Cross, Second Class: Matron Kate Fenwick, Sister Ursula Barclay, Miss Euphemia Philp, and Mrs. Edith Williams, B.R.C.S. Also five V.A.D. nurses. Military Medal: Miss Josephine Pennell, V.A.D. All these ladies were subsequently received at Marlborough House by Queen Alexandra.

At the Investiture held on June 14 the King personally conferred the decoration of the Royal Red Cross on the following members of the nursing services: First Class, Asst. Matron Jane Paul, Q.A.I.M.N.S.R.; Sister Isabella Muir, T.F.N.S. Second Class, Staff-nurse Eliza Forrest, Q.A.I.M.N.S.R.; Miss Mary Hays and Sister Lucy Hurlston, B.R.C.S.; Miss Annie Sandle, V.A.D. His Majesty conferred the Military Medal on Miss Evelyn Gordon-Brown, First-Aid N.Y. All these ladies were subsequently received at Marlborough House by Queen Alexandra.

---

Visitors to the Royal Academy should not omit to look for Miss Eleanor Ross's telling portrait of Miss Edith Cavell. It was painted with a view to being presented to the Edith Cavell Homes of Rest for Nurses, and reproductions are being sold for

**ROUND THE HOSPITALS—*(continued).***

the benefit of the Fund at its offices, 25 Victoria Street, S.W. 1. Queen Alexandra has graciously accepted a replica of the portrait.

The Committee of the Nation's Fund for Nurses together with a number of other guests were entertained by Mrs. Lionel Haring on June 11 at 4 Upper Grosvenor Street, and some interesting speeches were made. Sir Arthur Stanley explained the objects for which the Nation's Fund was founded and the causes which made an adequate endowment of the College of Nursing indispensable to the profession. He made it clear to his audience that the need for helping individual nurses was acute. Cases of distress were increasing daily and their seriousness and sadness were increasing also. He had lately sent to Queen Alexandra a short statement of some of the cases in which relief was being given. If the public could be made acquainted with them they would at once recognise the need for contributing funds. In all probability the need will still further increase when at the signing of peace hospitals are demobilised. Sir Arthur stated that the College had already obtained half its endowment, and that he saw some of the remaining half in the near future. This is excellent news.

The Association of Hospital Matrons will hold its first quarterly meeting on Saturday, June 28, 1919, at 3.30 p.m., at the Medical Society's Rooms, Chandos Street, Cavendish Square, W. Miss McDonald, R.R.C., Matron-in-Chief Canadian Army Nursing Service, will read a short paper on her experiences in hospitals overseas and at home. Mr. Leonard Lyle, M.P., it is hoped, will speak on the present position of the two Bills for State Registration, now before Parliament. Tea will be served after the meeting.

Captain Elliott is reported as stating at the meeting of the Nation's Fund for Nurses that nurses are called on to work from sixty-nine to eighty-four hours a week, and that the nation did not realise there were some who worked twelve hours a day every day in the week. The highest number of hours of duty shown in the returns sent in to the Salaries Committee of the College of Nursing was seventy-one hours a week on day duty and eighty-four hours a week on night duty, so that the speaker was well within the facts. Wide publicity has been given to these statements in the lay Press, and we attach much importance to their being pressed on the man in the street, who is, after all, the largest employer of nurses in the country. It is astonishing that the just claims of nurses have never been ventilated on the occasion of municipal elections, and that the majority of subscribers to voluntary institutions which depend for their existence on the maintenance of an efficient nursing staff are wholly indifferent to the conditions under which the nurses pursue their duties. We fear that improvement will only come very slowly, unless jogged either by State intervention or by a professional organisation powerful enough to refuse official sanction to institutions maintaining these abuses.

The National Birth-Rate Commission has had no more stimulating witness to examine than Professor Leonard Hill, of the Medical Research Committee, who gave evidence last week on the causes of high mortality among infants. His own experimental work, carried out during many years, has demonstrated that it is not so much the chemical impurities in the air as the physical properties, the heat, moisture, and close feel in an ill-ventilated room which depresses vitality. Ignorant mothers surround their infants with a tropical, still, humid atmosphere, which checks the cooling and evaporative effects on the skin, and brings on digestive, nutritive, and respiratory troubles. Lack of windage is, in fact, the root cause which accounts for the high infant death-rate in crowded quarters. The ray of hope which this discovery brings to nurses working in slum quarters will be at once appreciated. They need not worry about the contamination of the air from sources beyond their control. They must concentrate on keeping a current of air always moving in the smallest, most crowded tenement apartment. The cool morning air which Professor Hill extols as the natural stimulus to activity and appetite, to deep breathing, active circulation, thorough oxygenation, and good digestion is within the reach of all. When rich and poor alike recognise its effects in provoking mental resistance to disease the battle will be won.

Both Victory and Funding Loans offer a splendid investment in every way suitable for women earning their own living. By payment of £5 down and subsequent payments of £10 a month until the sum of £85 in the one case and £80 in the other has been completed, stock to the value of £100 at 4 per cent. interest can be purchased, and in addition to the value of the security the owner will have the consciousness of contributing so far as lies in her power to the consolidation of victory. If only the war gratuities so long overdue could be paid up this month there would be every inducement to sisters to make the fruits of their labours secure by this easy means. The terms of the loan should be fastened up in every nursing institute. They appeal with great force to private nurses in good work handling big cheques every month while provided with everything they need.

As we go to press the College of Nursing is holding its first provincial annual meeting in Manchester. Anxieties about weather have forsaken the promoters of meetings this season, and a fine attendance, as well as a fine day, seem to be assured. Few more interesting events have taken place in the short life of the College. The occasion should call forth a spontaneous and unanimous response from nurses all over the country. It will not be the first time that Manchester has given the lead to England.

**ROUND THE HOSPITALS—(continued).**

The Bristol Centre of the College of Nursing, Limited, held its first annual general meeting on June 12. Miss Baillie was in the Chair. Miss Densham, Hon. Treasurer, was unfortunately unable to be present. Miss McHardy, Hon. Secretary, read the report of the year's work. Miss McHardy then read, at the Hon. Treasurer's request, her financial report, which showed the excellent balance in hand of £27 17*s.* 6*d.* At the conclusion the local members voted for the officers of the centre for the new year, April 1, 1919-1920, and re-elected all those who had served in the previous year: Miss Baillie, Local Representative; Miss Densham, Hon. Treasurer; Miss McHardy, Hon. Secretary; Miss White, Hon. Asst.-Secretary. These ladies command the confidence of the whole centre.

The annual report of the Bristol Centre shows that the wish expressed on its formation that it might be truly represented and sum up the full interest of the nursing life of the City and surrounding country, has been remarkably fulfilled. The Committee as it now stands represents all the British institutions; it includes the military, special and Poor-Law institutions in the districts from Taunton to Cheltenham. It is, therefore, entitled to speak with great authority on all matters affecting the interests of its members, and we observe with interest that it has attracted the support of the local members of Parliament, Sir William Howell Davies and Colonel Gibbs, who have promised their consideration in dealing with the proposals for legislation now before Parliament. The lectures given by the Bristol Centre during the past year have been of a striking character and have attracted great attention. The Finance Committee has enlisted the services of leading Bristol citizens and has done admirable work for the Nation's Fund for Nurses. The membership roll shows 122 members, a most encouraging record for a first year.

They believe in Australia that the "democratic outlook on life" of the Australian nurses puzzled the British nurses. Some one heard a horror-stricken Englishwoman complaining that one of the Australian Sisters was seen walking in the street with an orderly, "who happened to be her brother." But it was not only in the Australian contingent that officers of quite humble position in civil life were to be encountered, nor do we think it would be difficult over here to parallel the Australian driver of a motor-lorry who "could easily write a cheque for £10,000." What we admired in the Australian nurses was not so much their democratic outlook—that is common enough—but their sense of duty. At the welcoming speech made by Matron Gould on the return of the War nurses she hit the nail on the head when she said, amid immense applause, "Of 119 nurses that I had on my books during two years I only had one disappointment. The New South Wales nurses are not angels—no, not angels—but they are very decent women." It must be acknowledged that her percentage of failures compares very favourably with that of Florence Nightingale.

Our readers will remember the Wittring Court Nursing Home, where Jessie Charlotte Spurling died last winter as a consequence of neglect. Mrs. Binstead, the head of the Home, was charged last week with manslaughter, in accordance with the verdict at the inquest, and was declared of unsound mind. She will be detained in an asylum. Her fellow-accused, Mr. Harry Hall, an estate agent of Southend, was sentenced to three months' imprisonment, with hard labour, at Chelmsford Assizes. He had been given a power of attorney by Mrs. Binstead, and was charged with having control of the Home and being guilty of such negligence as accelerated Miss Spurling's death. Mr. Justice Horridge, in passing sentence, commented severely on the prisoner's conduct. It was clear that he was told the patients were in need of medical attendance and nursing, and that he neglected his plain and obvious duty. That such a place should exist was almost incredible. We look to the Ministry of Health to see to it shortly that such abuses are rendered impossible evermore in this country.

An important article on "School Nurses" in *The Times Educational Supplement* of June 12 cordially endorses the conclusions arrived at by the Salaries Committee that the trained nurse of a school should receive the same minimum salary as the trained teacher under the same employing council. It was recommended that the average salary of the health visitor, found to be £75 to £120 non-resident, should be raised to a minimum of £120 with or £130 without uniform, and the writer of this article thinks that the school nurse ought not to rank lower than the health visitor or be worse paid. The design of Mr. Spurley Hey is to get the whole care of the child under the direction of the Board of Education. In that case school nurse and health visitor would be one and the same individual, continuing her ministrations from the infant in nursery, school, and crêche to the older child in the elementary schools. It is undoubtedly true that the more dignified position (as it seems to be thought) and the higher salary accorded to health visitors by no means indicate that the school nurse working in the same place is less competent or less highly trained. We fear it means that trained nurses are more easily "put upon" than other women workers.

The new type of airships projected for the cure of tuberculosis patients will demand a new type of aerial nurse. If the treatment proves successful, and there are grounds for believing it may stimulate re-action in certain selected cases, some form of training in air-ships conditions will become a feature in Hospitals for Consumption. We are confident this branch of work will not lack many enthusiastic volunteers.

